# Retinal detachment following focal laser to ruptured retinal artery macro aneurysm

**DOI:** 10.22336/rjo.2021.13

**Published:** 2021

**Authors:** Avadhesh Oli, Divya Balakrishnan

**Affiliations:** * Smt. Kanuri Santhamma Centre for Vitreoretinal Diseases, LV Prasad Eye Institute, Hyderabad India

**Keywords:** retinal detachment, focal laser, retinal arterial macroaneurysm

## Abstract

A 53-year-old lady presented with inferior retinal detachment (RD) following focal laser for retinal artery macroaneurysm (RAM). She underwent focal laser with intravitreal gas injection elsewhere; however, no retinal break was localized on the examination. The patient was taken up for vitreoretinal surgery. Intraoperatively, it was noted that the retinal detachment was not extending to the retinal periphery and primary retinal break was not localized even during the scleral depression. Under high magnification, using a macular lens, a slit-like retinal break was noted at the area of previous focal laser. Focal laser for RAM probably caused this retinal break leading to RD.

The clinician needs to be aware that during focal laser of ruptured RAM, haemorrhage may preclude the view of retinal structures leading to inadvertent use of excessive laser energy. Retinal breaks may form at the site of laser due to coagulative necrosis. During surgical management of RD in such cases, the area of focal laser should be thoroughly examined under high magnification to avoid missed breaks.

## Introduction

Retinal artery macroaneurysm (RAM) is an acquired focal retinal artery dilatation in the first three orders of retinal vessel bifurcations. RAM may be misdiagnosed initially, due to various clinical presentations. The long-term visual outcomes, even without treatment, are reported to be favorable. However, laser photocoagulation of macroaneurysm and anti-vascular endothelial growth factors (VEGF) agents to treat the exudation are commonly used [**1**]. If the RAM has already ruptured, it closes spontaneously due to thrombosis and does not require treatment [**2**]. 

We present an unusual case of ruptured RAM of a female patient who underwent focal laser elsewhere and subsequently presented after two weeks with inferior retinal detachment (RD). The challenges in the management and clinical course of the case are discussed in this report.

## Case presentation

**Fig. 1:**
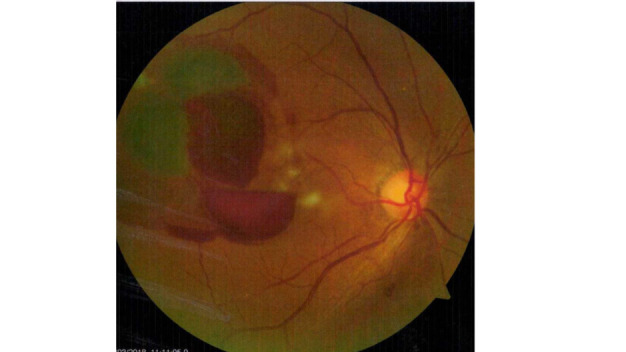
Fundus photo showing a ruptured RAM with haemorrhage at all levels

**Fig. 2:**
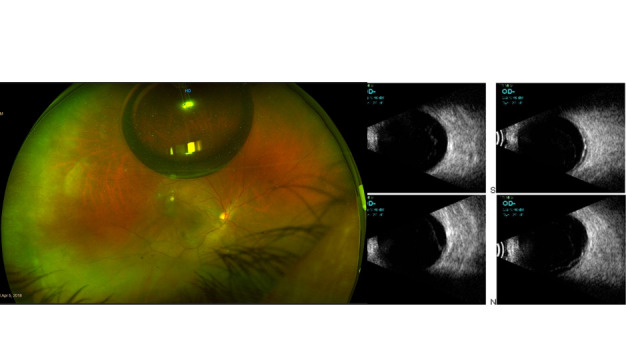
**A** Optos wide-field fundus photography showed inferior RD with laser scars temporal to the fovea with a small residual gas bubble; **B** Ultrasound B scan showed shallow retinal detachment

A 53-year-old woman presented with a decrease in vision in the right eye (RE) of one-month duration, for which she underwent some laser procedure elsewhere. Her visual acuity was 20/ 125 in RE and 20/ 20 in the left eye (LE). Anterior segment examination was unremarkable except for early cataract with nuclear sclerosis grade I in both the eyes. Fundus evaluation showed inferior RD in the RE with pigmented chorioretinal atrophic (CRA) areas in the inferior periphery and focal laser marks superotemporal to the fovea, adjacent to vessel arcade. A small gas bubble was noted in the vitreous cavity superiorly. She was diagnosed with RE rhegmatogenous retinal detachment. The ultrasound B scan confirmed shallow inferior RD and she was advised to undergo vitreoretinal surgery. On the review of available old documents, a ruptured RAM with haemorrhage at all levels in the retina was confirmed (**Fig. 1**). She already underwent focal laser and intravitreal gas injection two weeks before (**Fig. 2 A,B**).

In the absence of clinically evident primary break, the rhegmatogenous RD was presumed to be due to the inferior chorioretinal atrophy (CRA) lesions. The exudative RD was ruled out due to absence of shifting fluid or convex configuration.

**Fig. 3:**
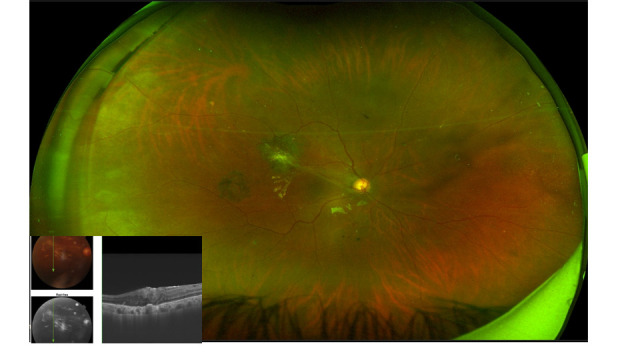
At final follow up, fundus photo showed attached retina and OCT showed epi-retinal membrane

She underwent 25-gauge pars plana vitrectomy. After posterior vitreous detachment (PVD) induction and completion of vitrectomy, retinal periphery was reexamined with scleral depression. However, primary retinal break could not be localized except for the inferior pigmented CRA. As the RD was not extending up to ora serrata temporally, the previous focal laser mark area was examined under high magnification using a macular lens. A small preretinal pigment clump adjacent to the superotemporal arcade at the edge of laser mark with a tiny break and schlieren was observed from that spot. The retina was settled by fluid air exchange, and silicone oil tamponade was used after endolaser. At final follow up after six months, she underwent silicone oil removal. The final visual acuity was 20/ 80 with the attached retina (**Fig. 3**).

## Discussion

Focal laser photocoagulation is used in cases of RAM for inducing thrombosis of the aneurysm. The typical spot size used is 100 microns, which delivers energy at a specific point. Excessive power may cause retinal breaks due to coagulative necrosis. In the presence of haemorrhage, if the view of the retina is precluded or if the laser spot is not focused correctly, retinal damage can occur. In this case, probably the ophthalmologist faced difficulty in focusing the laser spot on the RAM lesion due to haemorrhage. He would have inadvertently increased the laser power with a small spot size leading to delivery of excessive laser energy. Although cases of RD following extensive peripheral laser have been sparsely reported, however, on an extensive literature search, we could not find any report on RD after focal laser. Few authors have used a subthreshold laser to avoid adverse effects associated with the conventional laser with comparable results [**3**]. Recent studies showed that such a case of a ruptured retinal macro aneurysm is best managed with intravitreal anti-VEGFs [**4**]. These drugs act by reducing the exudation, however, there is no fixed treatment protocol. Pharmacotherapy may reduce the complications associated with laser treatment. In most of the reported cases in literature, the macular edema and RAM regressed with intravitreal injections [**5**].

## Conclusion

Laser-induced breaks leading to retinal detachment should always be kept in mind in a patient who underwent focal laser treatment. Retinal detachment due to posterior pole breaks may not extend until the ora serrata, unlike peripheral breaks, hence the area of retinal laser should be examined meticulously with a macular lens under high magnification to look for laser-induced breaks. 

**Conflict of Interest**

Authors state no conflict of interest.

**Informed Consent and Human and Animal Rights statements**

Informed consent has been obtained from all individuals included in this study.

**Authorization for the use of human subjects**

Ethical approval: The research related to human use complies with all the relevant national regulations, institutional policies, is in accordance with the tenets of the Helsinki Declaration, and has been approved by the institutional review board/ committee of Smt. Kanuri Santhamma Centre for Vitreoretinal Diseases, LV Prasad Eye Institute, India.

**Acknowledgements**

None.

**Sources of Funding**

None.

**Disclosures**

None.
